# Characterization of Chilean hot spring-origin *Staphylococcus* sp. BSP3 produced exopolysaccharide as biological additive

**DOI:** 10.1007/s13659-024-00436-0

**Published:** 2024-02-04

**Authors:** Srijan Banerjee, Gustavo Cabrera-Barjas, Jaime Tapia, João Paulo Fabi, Cedric Delattre, Aparna Banerjee

**Affiliations:** 1 Instituto de Química de Recursos Naturales, Universidad de Talca, CP 3460000 Talca, Chile; 2 Universidad San Sebastián Campus Las Tres Pascualas, Facultad de Ciencias Para el Cuidado de la Salud, Lientur 1457, CP 4080871 Concepción, Chile; 3 Department of Food Science and Experimental Nutrition, School of Pharmaceutical Sciences, University of São Paulo, São Paulo, SP Brazil; 4 Food Research Center (FoRC), CePID-FAPESP (Research, Innovation and Dissemination Centers, São Paulo Research Foundation), São Paulo, SP Brazil; 5 Université Clermont Auvergne, Clermont Auvergne INP, CNRS, Institut Pascal, 63000 Clermont-Ferrand, France; 6 Institut Universitaire de France (IUF), 1 Rue Descartes, 75005 Paris, France; 7 Instituto de Ciencias Aplicadas, Facultad de Ingeniería, Universidad Autónoma de Chile, CP 3467987 Talca, Chile

**Keywords:** *Staphylococcus*, Hot spring, Exopolysaccharides, Structural characterization, Flocculation, Antioxidant activity

## Abstract

A type of high molecular weight bioactive polymers called exopolysaccharides (EPS) are produced by thermophiles, the extremophilic microbes that thrive in acidic environmental conditions of hot springs with excessively warm temperatures. Over time, EPS became important as natural biotechnological additives because of their noncytotoxic, emulsifying, antioxidant, or immunostimulant activities. In this article, we unravelled a new EPS produced by *Staphylococcus* sp. BSP3 from an acidic (pH 6.03) San Pedro hot spring (38.1 °C) located in the central Andean mountains in Chile. Several physicochemical techniques were performed to characterize the EPS structure including Scanning electron microscopy–energy dispersive X-ray spectroscopy (SEM–EDS), Atomic Force Microscopy (AFM), High-Performance Liquid Chromatography (HPLC), Gel permeation chromatography (GPC), Fourier Transform Infrared Spectroscopy (FTIR), 1D Nuclear Magnetic Resonance (NMR), and Thermogravimetric analysis (TGA). It was confirmed that the amorphous surface of the BSP3 EPS, composed of rough pillar-like nanostructures, is evenly distributed. The main EPS monosaccharide constituents were mannose (72%), glucose (24%) and galactose (4%). Also, it is a medium molecular weight (43.7 kDa) heteropolysaccharide. NMR spectroscopy demonstrated the presence of a [→ 6)-⍺-d-Man*p*-(1 → 6)-⍺-d-Man*p*-(1 →] backbone 2-*O* substituted with 1-⍺-d-Man*p*. A high thermal stability of EPS (287 °C) was confirmed by TGA analysis. Emulsification, antioxidant, flocculation, water-holding (WHC), and oil-holding (OHC) capacities are   also studied for biotechnological industry applications. The results demonstrated that BSP3 EPS could be used as a biodegradable material for different purposes, like flocculation and natural additives in product formulation.

**Graphical Abstract:**

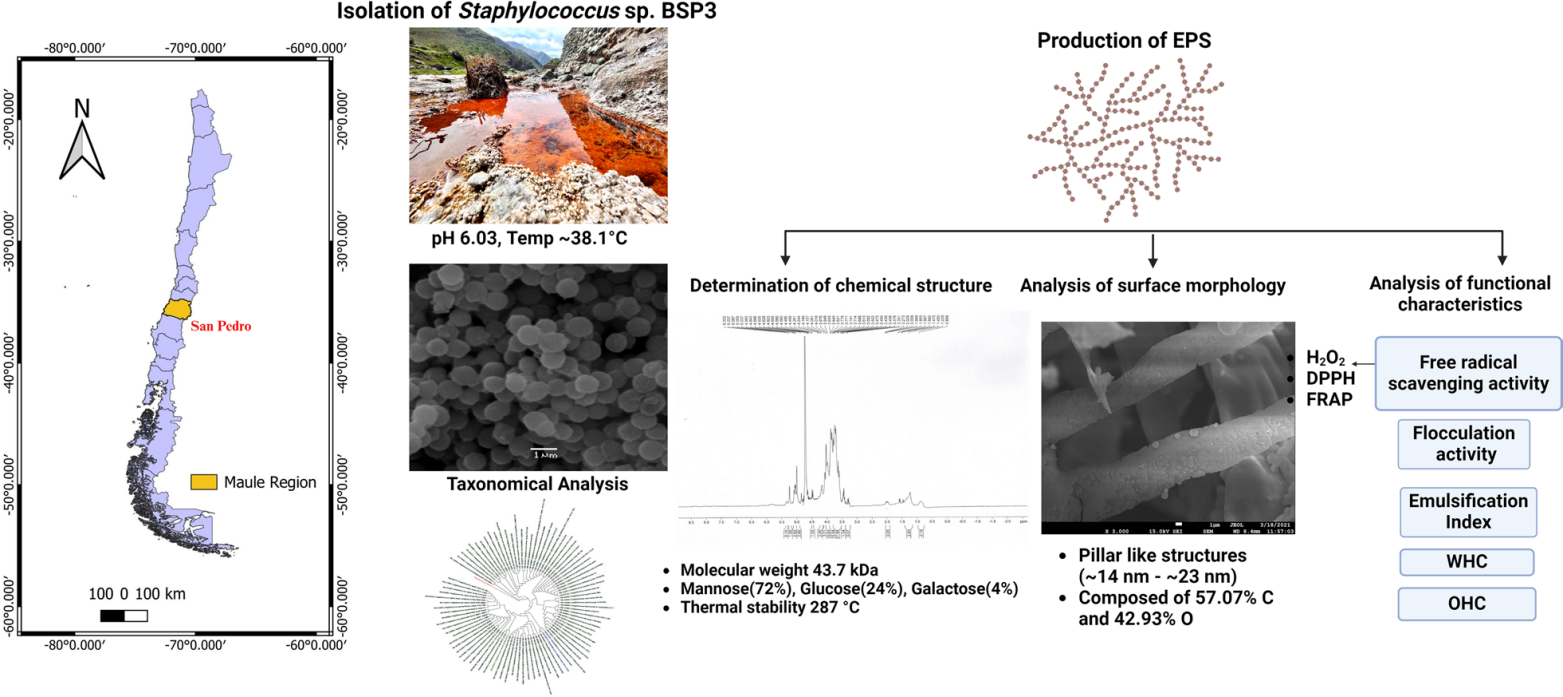

**Supplementary Information:**

The online version contains supplementary material available at 10.1007/s13659-024-00436-0.

## Introduction

In the present scenario, exopolysaccharides (EPS) derived from microorganisms gained very high importance in biotechnological industries as the trend is shifted by swapping artificial additives with bioactive compounds of natural origins [1–3]. The main reason is that they act as bioemulsifiers, which can stabilize the emulsion effect between hydrophobic compounds and water. In terms of nontoxicity, environmental compatibility, biodegradability, and selectivity, these bioactive compounds are more favorable than artificial additives [4]. Due to these properties, along with antiviral, antitumor, and immunoregulatory responses, EPSs also get attention in biomedical, biomaterials, cosmetics, and wastewater treatment industries [5–7]. EPSs derived from *Salipiger mucosus* are already well reported to have bio-removal activity against toxic metals in polluted wastewater environments which can be used to treat industrial effluents with heavy metal contamination [8, 9]. EPS produced by one halophilic bacterium *Halomonas almeriensis* also gained importance for its absorption capability of heavy metals like Pb, Cu or Co [9]. Scientists already reported Cyanobacterial EPS's role in its contribution in soil improvement by enhancing water retention, soil structure, nutrient availability, erosion control, microbial activity, pH stabilization, stress tolerance in plants with the formation of biological crusts [10]. Microbial exopolysaccharides are also well investigated for their potential in enhanced oil recovery [11]. EPS produced by *Azotobacter vinelandii*, *Xanthomonas campestris*, *Leuconostoc mesenteriodes, Streptococcus mutans* are also used as excipient in formulation of skin and cosmetics product as thickening, gelling, skin moisturizing or smoothing agents [12–14]. EPS derived from thermophiles are also well studied for several application related to these industries as these bacteria can adapt in a large set of temperature, pH and salinity and pressure [15–18].

Unlike mesophilic bacterial communities, a diverse range of bacteria can thrive in multiple extreme environments i.e., extreme temperature, high salinity, very low or high pH, extreme pressure, drought, exposure of high UV radiation. Sometimes they are also adaptable in trace metal deposit and toxic compounds [19–26]. For adaptation in these harsh environments, they accumulate EPS on their cell surface to stabilize the cell membrane structure. Their EPS structures are also altered based on the parameters of the harsh environments in which they thrive [27, 28]. Although EPS yield from thermophiles is normally less than mesophilic bacterial EPS, they have more importance in various industries because of their high thermal stability and low cytotoxicity. EPS produced by thermophiles can also be an alternative resource of artificial antioxidants used in the pharmaceutical industry for increasing the shelf life [29]. As reported by Wang et al. two EPS isolated from *Geobacillus* sp. strain WSUCF1 showed significant antioxidant activities and demonstrated high thermal stability of 319 °C and 314 °C [30]. In a previous report, the EPS produced by avirulent *Bacillus anthracis* PFAB2 isolated from an Indian hot spring showed better emulsification activity in olive, sesame, and rice oil when it is compared to commercial surfactant Triton X-100 [31]. In one of our previous study the EPS produced by *Bacillus haynesii* CamB6 isolated from a Chilean hot spring exhibit better flocculation activity and oil holding capacity than most popular commercially used bacterial biopolymer Xanthan gum [32].

Geysers, salt flats, and hot springs located in Altiplano and Atacama Desert, cold mountains and ice lakes of Andes Mountain, ice fields, lakes and fjords of Patagonia are the main extreme biotopes of Chile. The microbial dark matter of these sites remains vastly unexplored [33, 34]. Biotic potential generated by thermophilic microorganisms of the unexplored, slightly acidic volcanic hot spring of San Pedro has been unraveled in this study. The physicochemical properties of EPS derived from isolated thermophilic *Staphylococcus* sp. was studied by different techniques like FTIR, TGA, SEM, GPC, sugar analysis and AFM. The functional analysis as flocculant material, water and oil holding capacity and antioxidant properties was also assessed. The structure and function analysis of the *Staphylococcus* sp. BSP3 EPS showed that it is a promising biotechnological natural additive of the future.

## Results

### Analysis of water sample

From San Pedro hot spring (35°08′ 12″ S, 70°28′ 36″ W) located in Romeral commune in the central Andean mountain of Central Chile (Maule region) the water samples were collected for this study. Surface water temperature of the study site was ~ 38.1 °C and hot spring water was slightly acidic in nature (pH 6.03). Other than that, several physiochemical parameters like DO, BOD, TDS, conductivity, total alkalinity, chlorides, color, turbidity, sulfates, nitrates, sulfates, Al, As, Cd, Cu, Cr, Fe, Mn, Mg, Hg, Ni, Pb, and Zn have been also studied. Detailed information of the physiochemical characteristics can be found in Additional file 1: Table S1.

### Taxonomic identification and isolation of the Bacteria

**Fig. 1:**
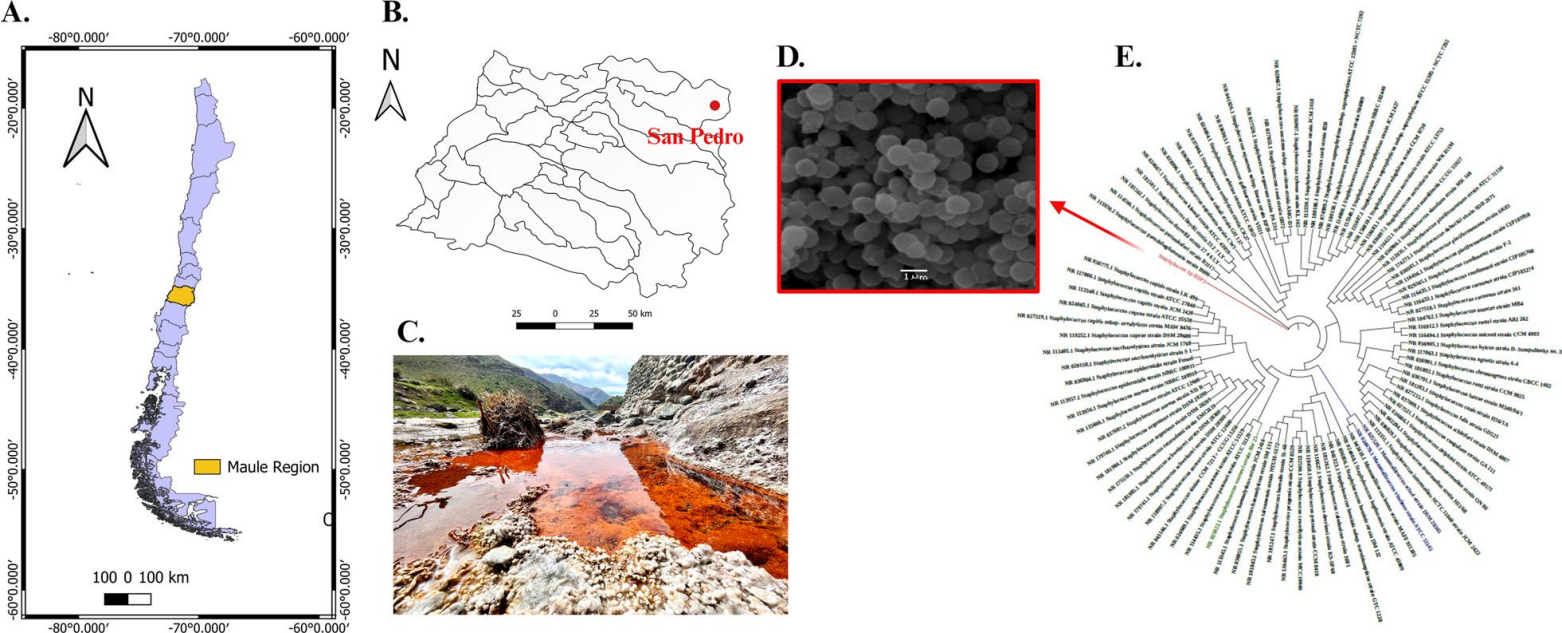
**A** Map image showing the Maule Region in Chile. **B** Map image showing the location of San Pedro hot spring in Romeral commune in Maule Region. **C** Photograph of the study site. **D** SEM image of the isolated *Staphylococcus* sp. BSP3 in 1 µm resolution. **E** Phylogenetic tree

Several thermophiles grew on the NA media at 38 °C. Out of them, white, convex, and opaque colonies of BSP3 have been initially isolated for EPS production. Nucleotide BLAST demonstrated that 16s rRNA sequence of BSP3 (GenBank accession no. OR915049) showed ~ 97% identity with 88 different *Staphylococcus* species. The phylogenetic tree is presented in Fig. 1E.

### Production and recovery of BSP3 EPS

Applying trichloroacetic acid precipitation method to get rid of proteins, followed by dialysis, the highest BSP3 EPS production has been achieved at 38 °C in stationary growth phase. The highest yield of BSP3 was 4.23 g L^−1^.

### Physiochemical characterization EPS

#### Analysis of molecular weight and monosaccharide composition

**Fig. 2:**
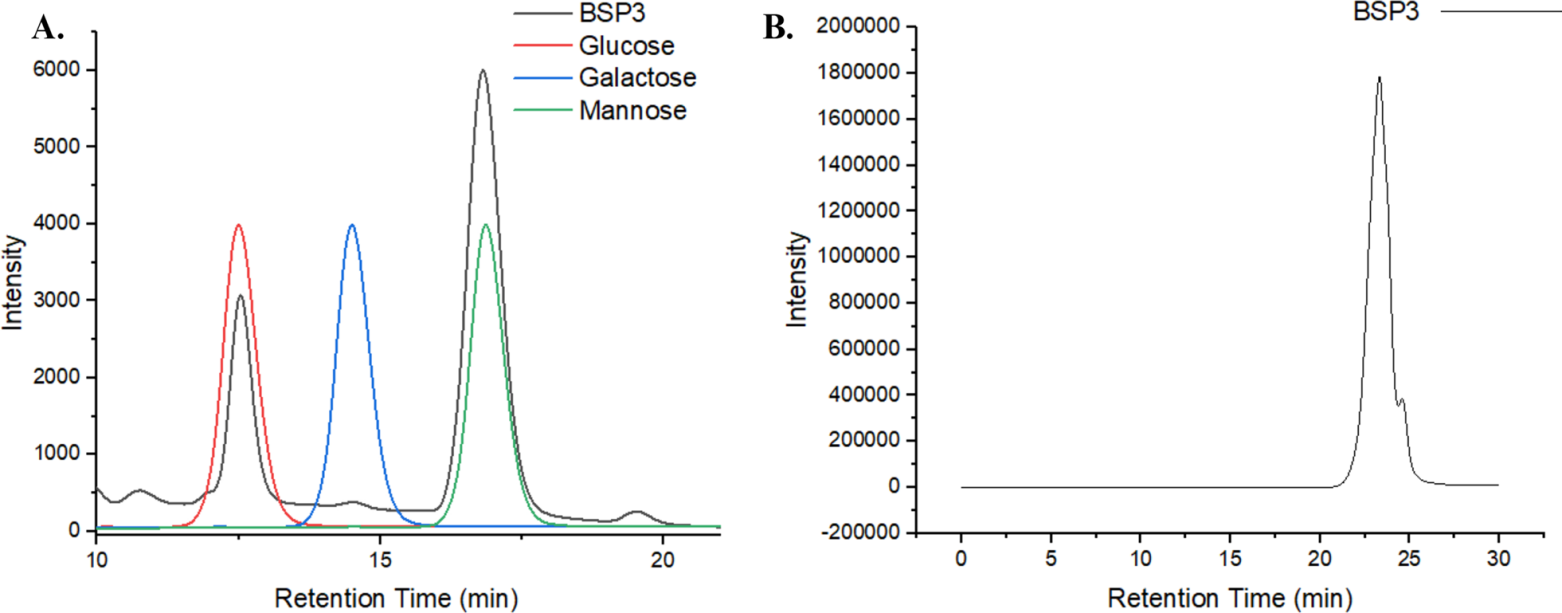
**A** HPLC chromatogram showing monosaccharide composition and (**B**) Gel permeation chromatogram showing the molecular weight of *Staphylococcus* sp. BSP3 exopolysaccharide

From monosaccharide HPLC analysis it was observed that the EPS produced by *Staphylococcus* sp. BSP3 is a heteropolymer chemically constituted by mannose (72%), glucose (24%), and galactose (4%) (Fig. 2A) residues in a molar ratio of 3:1:0.1, respectively. No other monosaccharides were detected using our method. The EPS average molecular weight (Mw) of 43.7 kDa has been also confirmed by GPC analysis (Fig. 2B).

#### Analysis of chemical structures

##### FTIR spectroscopy

**Fig. 3:**
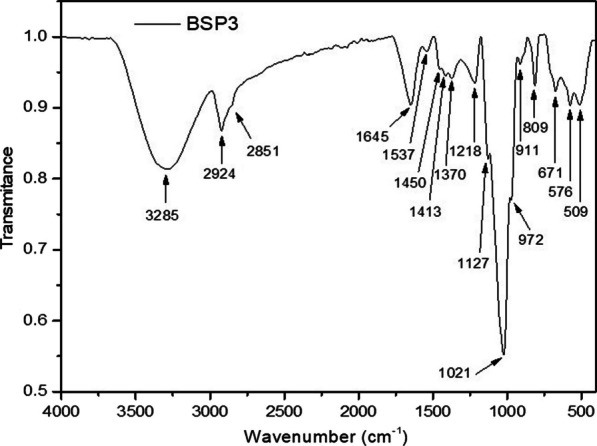
FTIR spectrum of *Staphylococcus* sp. BSP3 exopolysaccharide

To determine the EPS chemical structure the FTIR analysis was carried out and the resulting spectrum is presented in Fig. 3. The technique allows to determine the main functional groups of *Staphylococcus sp*. BSP3 exopolysaccharide. In above spectrum, it is observed a broad brand centered at 3285 cm^−1^ that is assigned as the stretching vibration of hydroxyl groups (ν –OH) from sugar residues [35]. Another peak at 2924 cm^−1^ corresponding to the stretching vibration of C–H bonds (ν–C–H) from the sugar ring. Protein traces in EPS can be identified from peaks at 1645 cm^−1^ and 1537 cm^−1^, due to the stretching vibration of carbonyl groups (ν C=O, Amide I) and (ν–C–N (C–N–H) + δNH, Amide II), respectively [36]. Other bands corresponding to C-H groups bond vibrations can be identified from peaks at 1413 cm^−1^ (δ–CH_2_) and 1370 cm^−1^ (δ –CH), respectively. Stretching vibration of glycosidic bonds (–C–C, –C–H, –C–O, –OH) and pyranosic rings are exhibited in the range 1200 to 900 cm^−1^ (ν_as_ C–O–C, ring). From the peaks ranging from 950 and 750 cm^−1^ stretching vibration of anomeric rings of exopolysaccharide was observed [37].

##### NMR analysis

**Fig. 4:**
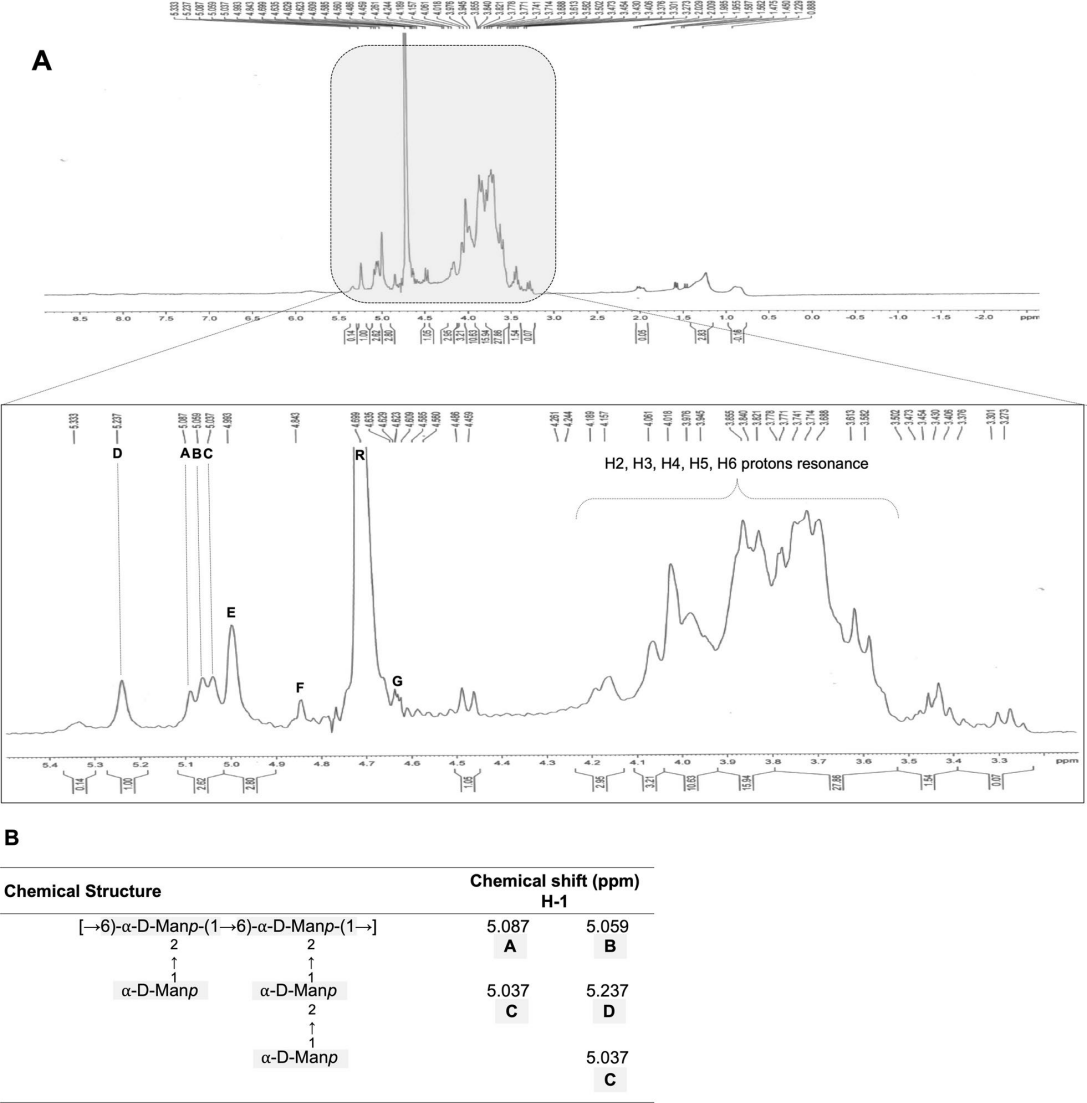
**A** ^1^H NMR spectrum of *Staphylococcus* sp. BSP3 exopolysaccharide (**B**) Partial chemical structure of the EPS

Generally, in the anomeric areas of carbohydrates, the α-configuration is observed within the range of 5.1–5.8 ppm (specifically for the anomeric proton), whereas the β-configuration tends to manifest in the 4.3–4.8 ppm range [38]. However, this principle is not universally applicable, particularly in cases of specific monosaccharides like mannose (Man) and rhamnose (Rha), where the coupling constants of the proton are comparably similar. Because of that, a thorough comparison to the literature must be done to validate the chemical shifts observed for both sugars. In our study, which used one-dimensional ^1^H NMR, the analyses were primarily given to the anomeric proton H1 since the region between 4.25–3.5 ppm is related to proton resonance of H2–H6, and because the EPS has an average molecular weight (Mw) of 43.7 kDa, determining the chemical shifts of H2-H6 protons is difficult due to superimposed values, as observed in Fig. 4A. Various studies on mannan characterization support primarily analyzing the anomeric proton H1, which has successfully assigned glycosidic linkages in these specific regions using more complex techniques.

Data from ^1^H NMR spectrum identified a set of chemical shifts related to d-mannopyranosyl. The non-reducing d-mannopyranosyl (R) was highly depicted at 4.699 ppm chemical shift. A set of α-d-mannopyranosyl (A, B, C, D) and β-d-mannopyranosyl (E, F, G) chemical shifts were assigned [39], demonstrating the EPS has a mixture of different glycosidic linkages.

According to the chemical shifts and the literature comparison, the isolated EPS seems to have a backbone of [→ 6)-⍺-d-Man*p*-(1 → 6)-⍺-d-Man*p*-(1 →] related to A and B values (Fig. 4B) [40–42]. Moreover, the backbone is 2-*O* substituted with 1-⍺-D-Man*p*, as observed by C and D chemical shifts [40–42]. These clear chemical shifts and data comparisons led us to infer a partial chemical structure of our EPS (Fig. 4B).

Regarding the β-d-mannopyranosyl chemical shifts, it was possible to identify a δ 4.843 value (F) and three δ values (4.623, 4.629, 4.635) related to a → 4)-β-Man*p*-(1 → unit [43, 44]. Together with the presence of a reducing end of β-d-mannopyranosyl chemical shift (E), the structure of the isolated EPS is more complex, and more experiments should be done to indicate the glycoside linkages beyond the 1 → 6)-⍺-d-Man*p* backbone with 2-*O* Man*p* ramifications. Furthermore, the anomeric proton signal at δ 5.333 is consistent with the phosphorylated mannose units [45], despite no traces of phosphorus being found in SEM–EDS analysis. This might be because NMR is more accurate, and the spectrum was only slightly detected.

##### Thermogravimetric analysis

**Fig. 5:**
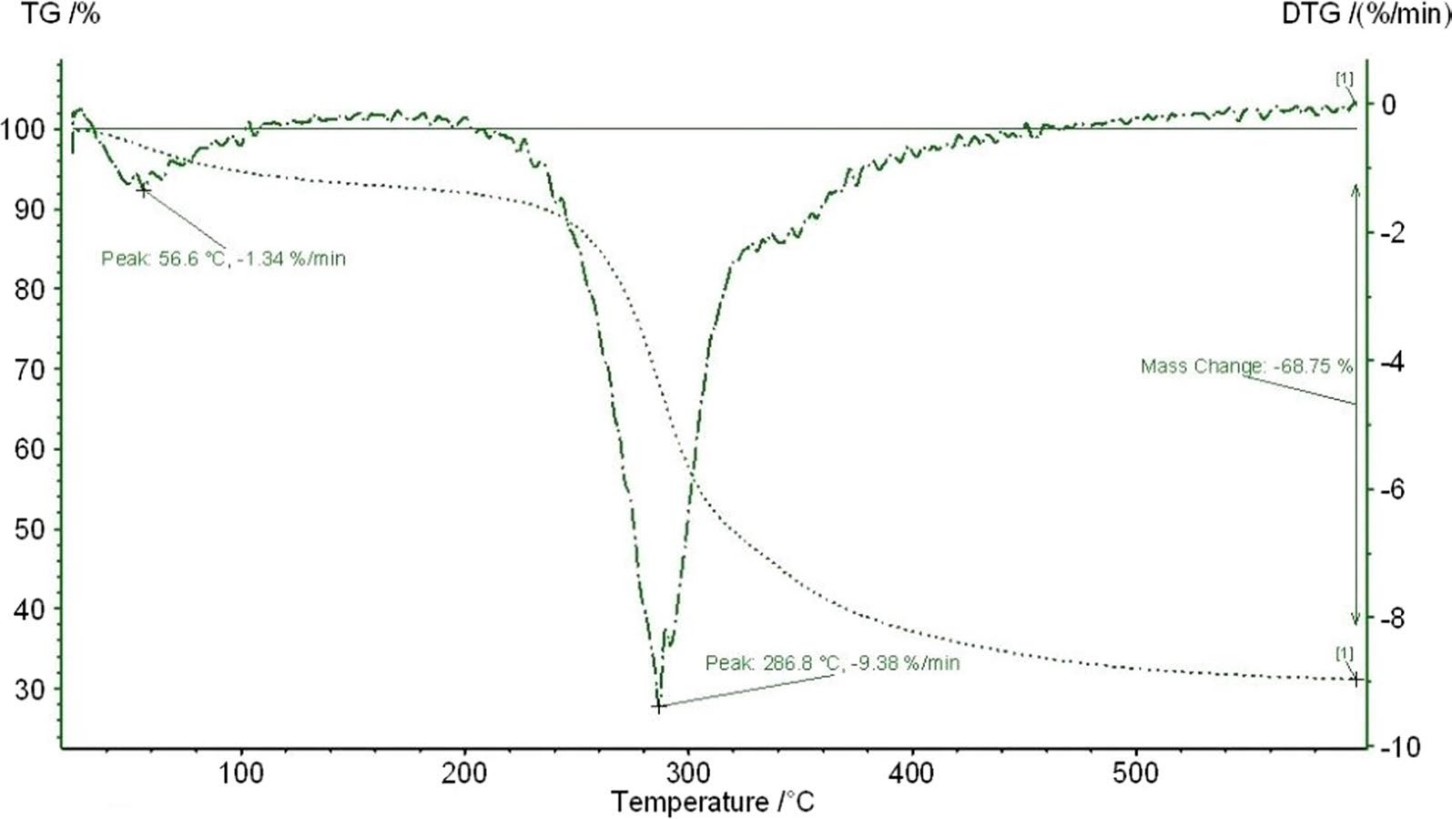
TG-DTG curves of thermophilic *Staphylococcus* sp. BSP3 exopolysaccharide

A thermogravimetric analysis was performed to understand the thermal stability of the *Staphylococcus* EPS. The thermal decomposition curves (TG and DTG) are presented in Fig. 5. It is observed to have a first thermal effect ranging from 34 to 112 °C, with a maximum decomposition rate temperature (T_peak_) at 59.9 °C, accounting for 6% mass loss. In this step, the EPS dehydration, due to unbound water release, takes place. In the temperature interval from 170 to 277 °C, the most significant thermal decomposition process of the EPS occurs. The maximum decomposition rate was peaking at 239.7 °C and accounting for 70% of mass loss. In this step, simultaneous and complex decomposition process takes place, including chain depolymerization and sugar dehydration. Finally, a third process ranging from 279 to 394 °C and peaking at 307 °C occurs. It is associated with a mass loss of 12% and could be due to the residual EPS decomposition. The above results showed the high thermal stability of *Staphylococcus* sp. BSP3 exopolysaccharide. According to the data, this EPS may be employed for functionalization and a variety of other chemical modifications to expand their potential application fields.

#### Analysis of surface morphology (SEM–EDS and AFM)

**Fig. 6:**
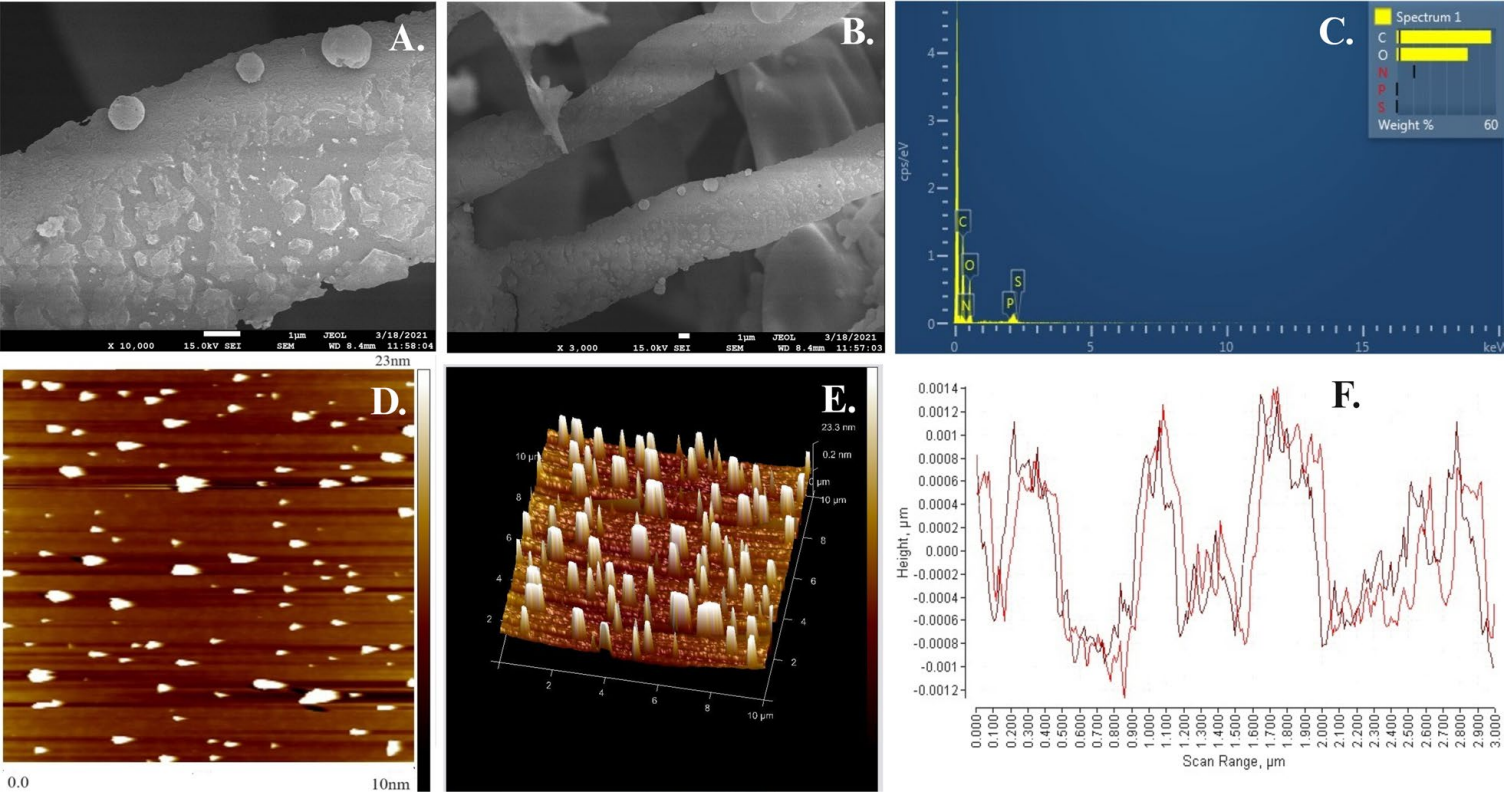
**A** Surface morphology of the BSP3 EPS where (**A**) × 3000 (**B**) × 10,000, respectively (**C**) SEM–EDS spectrum; **D** 2D and (**E**) 3D AFM images and (**F**) size distribution pattern

SEM–EDS and AFM have been performed to understand the three-dimensional structure and surface morphology of the BSP3 EPS. At 3000 × , 5000 × , and 10,000 × magnification range the SEM analysis has been executed. A nonporous, rough, compact surface has been observed. Upon further magnification it was identified that roughness is composed of unevenly distributed spherical appendages Fig. 6A, B.

SEM–EDS analysis is a widely used microscopic technique to understand the elemental distribution and percentages of each element of the EPSs. BSP3 EPS revealed that this biopolymer mostly composed of 57.07% carbon and 42.93% of oxygen of its total weight. No traces of nitrogen, sulfur or phosphorus were found for this EPS (Fig. 6C).

AFM is a widely used three-dimensional topographic technique which is based on the interaction between the atom of the sample surface and the sharp tip [46]. In our study 2D topographical images of AFM (Fig. 6D) confirmed that those uneven spherical appendages are of many different lengths. From 3D topographical images it is also observed that these spherical appendages have pillar like nanostructures ranging between ~ 14 nm and ~ 23 nm (Fig. 6E, F).

### Functional characteristics of EPS

#### Determination of antioxidant activity

**Fig. 7:**
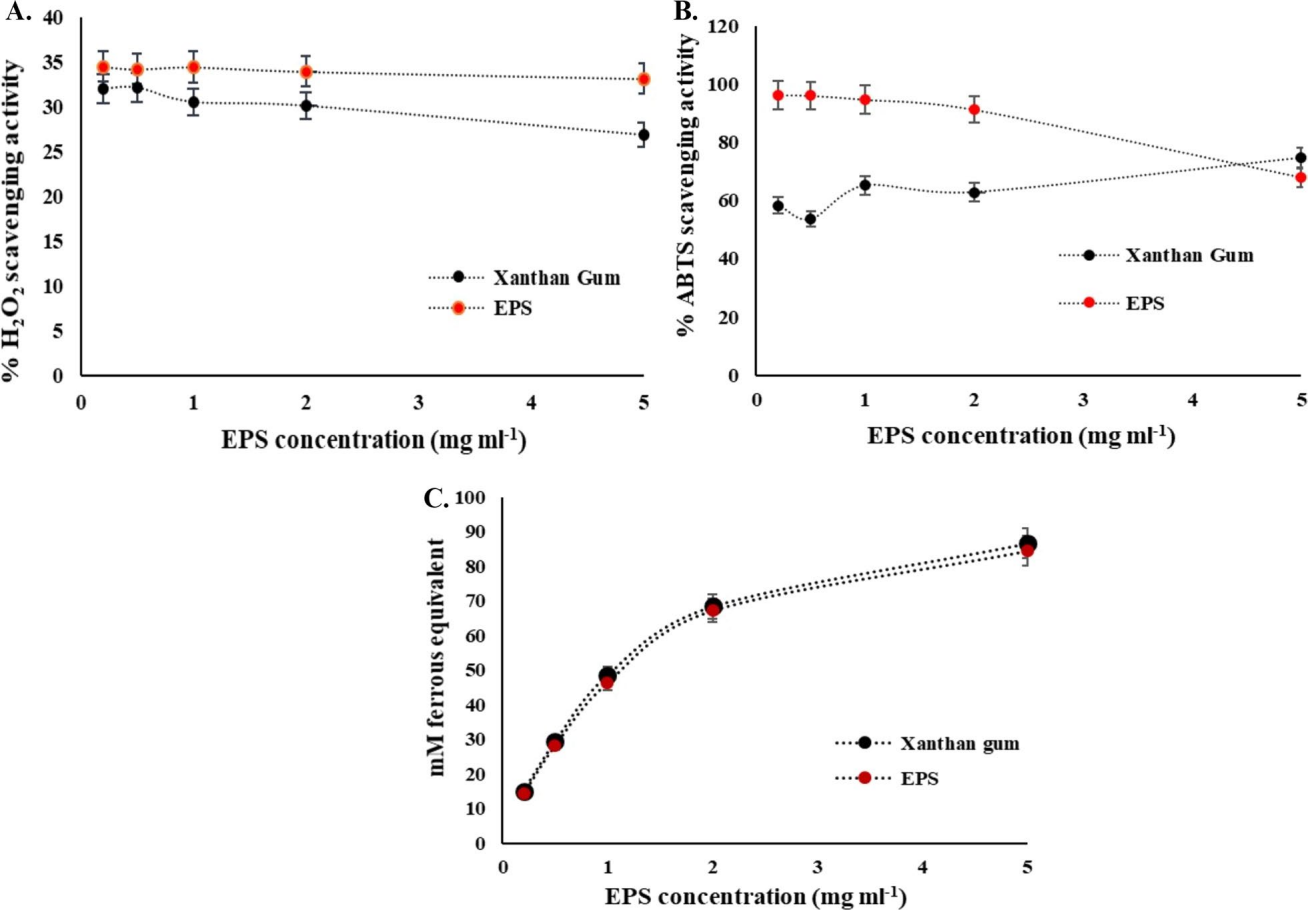
In vitro antioxidant capability of the EPS produced by *Staphylococcus* sp. BSP3; where (**A**) H_2_O_2_ radical scavenging activity; **B** ABTS radical scavenging activity; **C** FRAP activity

H_2_O_2_ and ABTS free radical scavenging activity and FRAP activity was performed for determination of in vitro antioxidant activity of our studied EPS and compared to commercially used bacterial EPS Xanthan gum (Fig. 7A–C). EPS produced by *Staphylococcus* sp. BSP3 performed better than Xanthan gum in all of the available concentration when H_2_O_2_ free radical scavenging activity was assayed. In 0.2 mg mL^−1^ concentration % of scavenging activity was highest for BSP3 EPS (34.46%). With higher concentration it got decreased. In available concentration higher % of scavenging activity of studied EPS with respect to xanthan gum was also observed in ABTS free radical scavenging activity assay except in 5 mg mL^−1^ concentration. With increasing EPS concentration % ABTS scavenging activity of BSP3 gradually decreased. In 0.2 mg mL^−1^ concentration BSP3 showed 96.16% ABTS scavenging activity which is 37.8% higher than Xanthan gum in this concentration. More or less similar FRAP activity has been exhibited by our studied EPS and xanthan gum which gradually increased with increasing concentration of Xanthan gum and BSP3 EPS.

#### Determination of flocculation activity

**Fig. 8:**
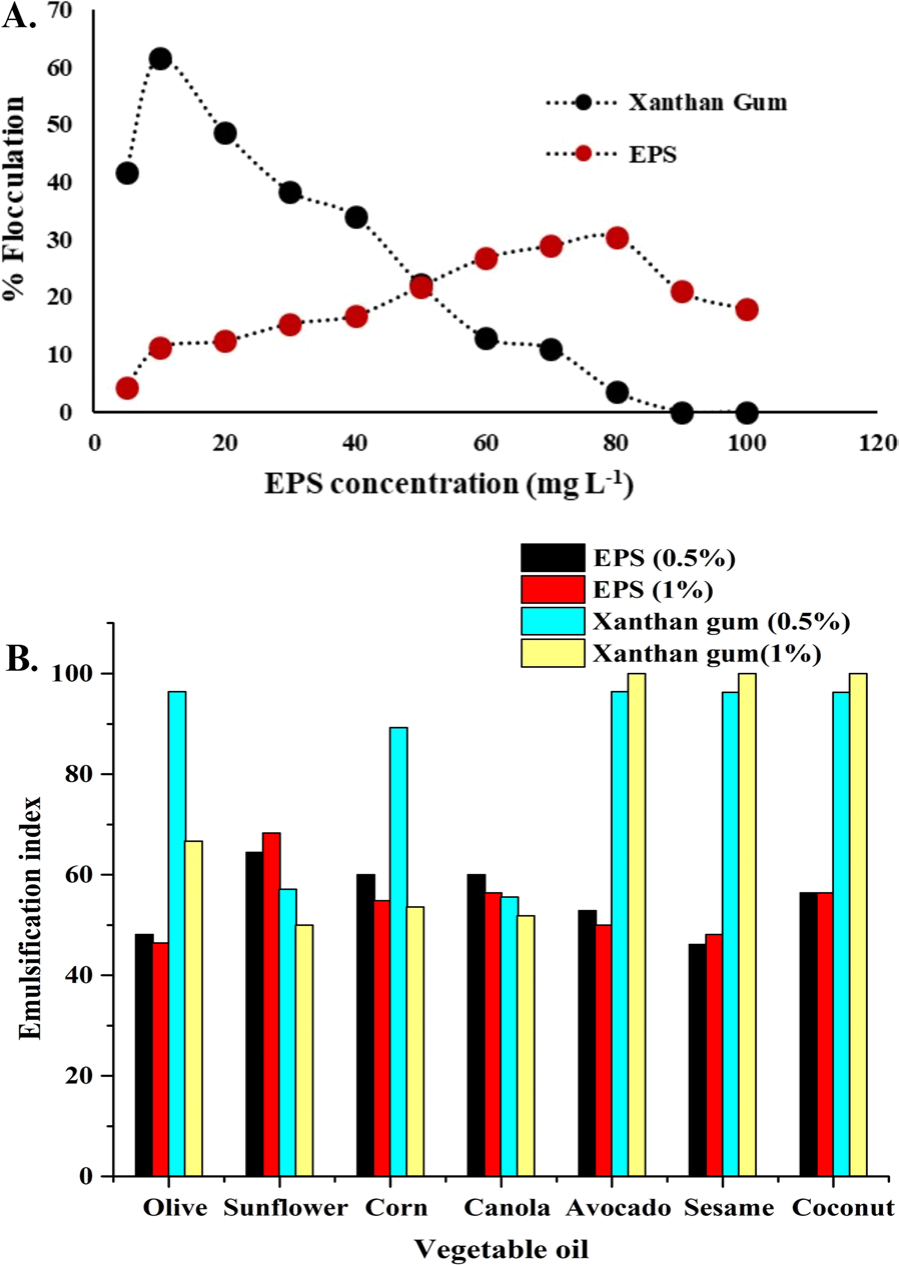
(**A**) Bioflocculation activity and (**B**) The emulsifying activity of the EPS produced by *Staphylococcus* sp. BSP3 in different vegetable oils compared to commercial EPS biopolymer

Kaolin clay suspension technique has been performed for flocculation activity analysis. From Fig. 8A it was observed that percentage of flocculation has been increased gradually till 80 mg L^−1^ concentration. Then percentage flocculation decreased with further increase with EPS concentration. Highest percentage flocculation 30.4% was observed at 80 mg L^−1^ EPS concentration and for lowest it was 17.8% in 100 mg L^−1^.

#### Analysis of emulsification index

Determination of emulsification activity of our EPS produced by *Staphylococcus* sp. BSP3 has been also compared with Xanthan gum (Merck). The studies were performed in two concentration (0.5% and 1%). Creating hydrophobic phase in different vegetables oils BSP3 EPS stabilizes them. When commercial bacterial EPS Xanthan Gum was compared to the study EPS from Fig. 8B it was observed that emulsification activity of the studied EPS was more stable in Sunflower oil (64.44) and Canola oil (60.00) and higher than Xanthan gum.

### Determination of OHC and WHC

111.3% Oil retention (OHC) has been shown by our study EPS. When statistical analysis has been performed using ANNOVA (only p value < 0.05 was selected), the result showed significant differences and greater stability compared to control Xanthan gum which showed 111.0% Oil retention. The water retention capacity of our study EPS was 102.6% where for Xanthan gum WHC was 183.3%.

## Discussion

This study specifically focuses on characterizing a novel functional EPS produced by thermophilic *Staphylococcus* sp. BSP3 isolated from the San Pedro hot spring in central Andean Mountain at Maule Region in Chile for application as biotechnological additives. Maule region Andean Mountain hot springs are very little explored and there has been no previous report on San Pedro hot spring of our study. The unique geographical locations of Chile consist with desert, salt flats, soda lakes, hot springs, geysers, fumaroles, permafrost, polar ice, acid mine drainage, ultra-violate exposition and volcanic rocks with trace metal contaminated soil provides a magnificent natural laboratory to study interactions of microbial dark matter thriving in theses extreme environments. There are several active and nonactive volcanos situated in central Andean Mountain nearer to our study site. Constant seismic movements were also reported in these regions. Previously scientist reported this type of geothermal water can have chemical signatures of both meteoric water and local magmatic reservoirs water circulation which is the case of Campanario hot spring situated in Laguna del Maule stratovolcano field [47, 48]. Flow of deep volcanic thermal water make this San Pedro hot spring as an “Acid spring”. High presence of sulphate (439.7 ± 1.8 mg L^−1^), chloride (13.93 ± 0.07 mg L^−1^) and fluoride (1.3 ± 0.0 mg L^−1^) in our water sample analysis are pointing towards the presence of volcanic arc beneath San Pedro. As observed in Additional file 1: Table S1A, metal deposits of Al, Cu, Mg, Mn, Ni, Pb, Zn also telling us about salinity of water and its interaction with sedimentary volcanoclastic rocks formed during the volcanic activity of the Andes’s active volcanos [49]. Microbial communities thriving in this various extreme environment with high salinity, temperature, trace metal deposition, over the last decade they are gaining attraction to the microbiologist as they produce value added substances for creating protecting sheaths to survive and communication with other microbial communities. Hot spring situated in central parts of Chile almost remained unexplored in terms of microbial niche and their compounds with bioactive potential.

*Staphylocooccus* sp. BSP3 isolated from San Pedro produced 4.23 g L^−1^ of EPS at 38 °C in nutrient media. According to previous reports it should be noted that thermophilic bacterium *Aeribacillus pallidus* 418 isolated from Sofia region, Rodopa mountain, west Bulgaria reported to have highest EPS yield of 0.066 g L^−1^ [7]. EPS yield of 0.114 g L^−1^ produced by from a thermophilic *Geobacillus tepidamans* isolated from another study site Velingrad hot spring, situated in Bulgaria was also reported [50]. The highest EPS yield of 0.00104 g L^−1^ produced by a thermophilic *Rhodothermus marinus* [51] and EPS yield of 0.0306 g L^−1^ produced by a thermophilic, endospore-forming isolate from the radioactive radon hot spring was also reported [36]. These results are much lower compared to the studied EPS. Although EPS produced by *Bacillus haynesii* CamB6 isolated from Campanario hot spring situated in central Andean Mountain of Chile produced a highest optimal EPS yield of 5.6 g L^−1^ at 55 °C [32]. So, our study EPS can be considered as a high yield.

An amorphous, irregular non-porous surface with pillar like nanostructures was identified by determination of surface morphology through SEM and AFM of the studied EPS. As mentioned from previous reports this compact structures with the films like feature denotes the strong interaction between hydroxyl groups (–OH) and water molecule of the EPS [52, 53]. Compact film like structure along with inter and intra molecular aggregation of biopolymers are reported before to have thickening property which can turn them a potential additive for biotechnological industries [53].

Previously, researchers reported that mannose, galactose, and glucose are the main constituents of the thermophilic bacterial EPS structure, which are isolated from hot springs [30, 32]. Although EPS structures of all of them are unique as the sugar constituents are present in different molar ratios. Sugar analysis and molecular weight analysis confirms that our medium molecular weight EPS mostly consist of mannose, glucose, and traces of galactose. The structure analysis confirmed a [→ 6)-⍺-d-Man*p*-(1 → 6)-⍺-d-Man*p*-(1 →] backbone 2-*O* substituted with 1-⍺-d-Man*p*. It has also been identified → 4)-β-Man*p*-(1 → units, but with no other chemical structure assessed. Thermal degradation of this EPS occurred in three steps providing the highest thermotolerance of 287 °C. This range of thermostability is very important as in biotechnological industries applications are set in wide temperature ranges. This type of mannan can be a biotechnological alternative to similar polysaccharides isolated from different agro-wastes sources [54]. In fact, it has a similar chemical composition, which could allow to be used as replacement of the later in some industrial applications. Also, biotechnological production will allow to obtain EPS with consistent and homogeneous composition between batch and to program production along year. In addition, the EPS production would be independent of the season, agricultural labor, edaphoclimatic conditions and not compete with food producing lands.

Mostly researcher reported about the bacterial polysaccharide intercellular adhesin (PIA), a polymer of β-(1 → 6)-linked glucosamine substituted with *N*-acetyl and *O*-succinyl constituents produced by other staphylococcus species like *Staphylococcus epidermidis* or *Staphylococcus aureus* which is the main constituents of the part of their extracellular matrix in biofilms and known to be responsible for biofilm associated infections and immune evasion [55, 56]. However, our studied EPS not only highly thermotolerant but its compact nature results in ropy characteristics. Ropy characteristics of additives are very important to build better texture in the biotechnological products [57]. Furthermore, some researchers reported that extremophilic bacterial EPS are suitable as emulsion stabilizer, gelling or suspending agents [58]. To determine the potential applications of the EPS, invitro antioxidant, bio-emulsion and flocculation capacity is assessed in this study.

Antioxidants are important in preservation of stem cells and tissues [59], cosmetics and personal care products [60] or stabilizing the active ingredients of pharmaceuticals [29], plastics and polymers [61] and petroleum and lubricants [62] as they can play vital role in inhibiting oxidation processes from reactive oxygen species (ROS). Regarding the antioxidant properties (H_2_O_2_, ABTS and FRAP) BSP3 EPS showed a concentration dependent outcome. % of scavenging activity for H_2_O_2_ and ABTS gradually decreased with higher concentration of EPS of BSP3 and Xanthan Gum. A 96.18% of free radical scavenging activity has been observed for BSP3 in very low concentration (0.2 mg L^−1^). It can be said that BSP3 outperformed Xanthan gum in context of H_2_O_2_ and ABTS scavenging activity. For FRAP activity mM ferrous equivalent increased along with increasing concentration of EPS. Both Xanthan gum and BSP3 performed similarly in this case. According to previous reports one of the vital causes of cells and tissue deterioration is lipid oxidation as lipids can be easily oxidized in presence of catalyst such as light, heat, enzymes, metals, metalloproteins and several microorganisms. Lipid oxidation in foods, cells and tissues and drugs, nutritional supplements can result in loss of fat-soluble vitamins and essential fatty acids. Inside the human body this oxidative stress generates destructive cellular effects which can cause several diseases [63]. The most effective way of controlling lipid oxidation is the use of antioxidants. In pharmaceutical industries the crucial role of excipients lies in modification of stability and rate of release of the drug to maintain expected pharmaceutical effects [64]. Antioxidants are introduced as excipients as they can improve the stability and shelf life of the active pharmaceutical ingredients. Cysteine, butylated hydroxianisole, propyl gallate, butylated hydroxytoluene, sodium metabisulfite are one of the most widely used antioxidants in pharmaceutical industries. These antioxidants are mostly made of polyhydric phenols or its derivatives. Previously scientists reported that these excipients are associated with numerous adverse effects diarrhea, gastric irritation, and tumors [65–67]. One of the solutions of this problem is using natural substances which provide adequate antioxidant activities. It can be said that antioxidant capacities of our studied EPS are functional at very low concentration.

In biotechnological application and also in chemistry industries EPS stabilized emulsions plays a crucial role as they can create macromolecular blocks in the aqueous solvents from the dispersed droplets using their thickening characteristics [68]. Previously, scientist reported bio emulsifier like emulsan, a polyanionic extracellular hetero-polysaccharide that are produced by *Acinetobacter calcoaceticus* RAG 1*, Acinetobacter venetianus* rag-1t ATCC 31012 and some *Streptomyces* strain that are widely used in treating skin infections and cream for skin protecting materials [69]. It can be said that our studied EPS BSP3 is efficient to stabilize oil in water and water in oil emulsions. It is to be noted that for canola oil our studied EPS performed better than commercial bacterial polymer Xanthan gum even in low concentration. Spatial stability of EPSs plays a crucial role in emulsifying the fatty acids forming extensive networks in the continuous phase [70].

Flocculating agents are immensely important in biotechnological industries for fermentation process as separating cells from the product is very easy with their aid [70]. Polysaccharides are known to be good bio-flocculating agent in pharmaceutical, nutritional supplements processing sectors, and flavor industry [71]. Bacterial EPS has charged groups as well as hydrophobic region in the polymer matrix that can create a bridge between contamination and cell to remove the contaminants in wastewater treatments [72]. Previously scientist reported applicability of bacterial EPS produced by thermotolerant strain *Bacillus* sp. ISTVK1 as a cost-effective bio-flocculant for wastewater treatments [73]. Stable flocculating activity was observed for our studied EPS. % flocculation was low in low EPS concentration, gradually it increases with increasing concentration of BSP3 EPS. Although after 80 mg L^−1^ concentration % flocculation decreased sharply. Presence of charged group in protein traces in initial concentration and highly viscous phase in more than optimal concentration can hamper the flocculation activity of EPSs [74, 75].

Our studied EPS BSP3 shows 111.3% OHC which is better than some previously reported oil retention capacities of EPSs produced by *Lactobacillus* (15.9%) [76] and *Weissella confusa* species (5.1%). Absorption of organic products to the surface of the substrates is indicated by OHC [77]. It depends on the chemical composition, porosity, and affinity of the biopolymer to oil. WHC of our studied EPS was also very high (183.3%) which is also higher than some previously reported EPSs isolated from *Lactobacillus* (8.95%) [76] and *B. licheniformis* (98.8%) [78]. Similar results was observed from EPS CamB6 produced by *Bacillus haynesii* isolated from Campanario hot spring in Central Chile (OHC 111.9%, WHC 102.9%) [32]. Although the percentages OHC and WHC of EPS Chitosan from animal origin is very much higher (WHC 135%, OHC 635%) [79].

Considering these results, it can be said that BSP3 EPS isolated from *Staphylococcus* sp. showed optimistic potential as natural emulsifying, antioxidant, and flocculating agent which can be used as promising natural additive in the field of biotechnology.

## Conclusions

Largely complex yet dynamic EPS substances fulfil many functional roles in bacterial cells ranging from adhesion of bacterial cells, cohesion of biofilms for surviving in extreme environmental condition to retention of water along with providing tolerance to host defenses or several microbial agents and many more. The findings of this study depict the use of EPS produced by *Staphylococcus* sp. BSP3 isolated from a previously unexplored Chilean hot spring San Pedro as a thermotolerant, antioxidant which can provide a large a set of applications as a potential biotechnological additive. Though halophilic *Staphylococcus* communities was already reported in poly extreme environments of Chile, to the best of our knowledge this study is the first to highlight a functional EPS produced by thermophilic *Staphylococcus* sp. BSP3 from Chilean hot springs.

## Materials and methods

### Analysis of water sample

From San Pedro hot spring located in in the Maule region of central Andean mountain of Chile (35°08′ 12″ S 70°28′ 36″ W) water samples were collected from a depth of at least 0.5 m. A pH/ORP meter from LAQUA PH120-K, HORIBA, Kyoto, Japan was used to measure the temperature, pH and conductivity of the water. Filters of 0.45 μm porosity have been used for the physiochemical analysis of the collected water using polycarbonate system. Analysis was executed using the protocol depicted by Standard Methods for the Examination of Water and Wastewater as described by INN-Chile [80]. High purity reagents from Suprapure, Merck, Darmstadt, Germany has been used. Dissolved oxygen (DO), biochemical, oxygen demand (BOD), total dissolved solids (TDS), total alkalinity, chlorides, color, turbidity, sulfates, and nitrates are measured to determine the physiochemical properties of the water sample. The Winkler method (Standard Methods for the Examination of Water and Wastewater) has been used to carry out the fixation of the water sample for determining the concentration of DO. In the Romeral commune near Chile and Argentina border where the San Pedro hot spring located in those volcanic fields aluminum (Al), arsenic (As), cadmium (Cd), copper (Cu), chromium (Cr), iron (Fe), zinc (Zn), manganese (Mn), magnesium (Mg), mercury (Hg), nickel (Ni), and lead (Pb) are the abundant metals. These metals are also detected from the samples. A Thermolyne Cimarec hot plate at 105 °C to a final volume of 50 mL was used for evaporation for performing the preconcentration of 500 mL filtered sample with 1 mL of 70% nitric acid except metals like Hg and As. Atomic absorption spectroscopy(AAS) (Perkin Elmer 1990, Norwalk, Conn, USA) was executed using a spectrophotometer from Thermo Fisher Scientific ICE 3000 Series, Cambridge, UK as previously described by Fierro et al. [81]. Cold vapor techniques and Hybrid evolution techniques were performed for detecting Hg and As, respectively. Detection limits of trace metals are mentioned in Additional file 1: Table S1.

### Taxonomic identification and isolation of the bacteria

After performing the serial dilution of the water sample, aseptically inoculation was executed with 50 μL of each dilution in nutrient agar media (NA, difco, pH 5.8). Taking into consideration that surface temperature of the San Pedro hot spring was observed 38.1 °C the inoculated media was incubated at 38 °C. After 72 h a white, convex, and opaque colonies of BSP3 was observed and then EPS productions are performed. For taxonomic identification, DNA of the bacterial colony was isolated and 16srRNA hypervariable regions was amplified with the universal primers 27F and 1492R. After sequencing the obtained amplicons, taxonomic identification was performed using the Nucleotide BLAST (https://blast.ncbi.nlm.nih.gov/Blast.cgi). A phylogenetic tree has been prepared using maximum likelihood score with the help of MEGAX software [82] after calculating the distances between sequences [83]. 16s rRNA sequence is deposited in GenBank.

### Production and recovery of the BSP3 EPS

To perform production of EPS bacterial cells during stationary phase were harvested in nutrient broth followed by treatment with 4% Trichlroacetic acid (TCA) (w/v) for 30 min as described by Rimada and Abraham [84]. Centrifugation at 4 °C, 5000×*g* for 20 min was applied to TCA precipitated cells to achieve protein precipitation. Chilled cell free supernatant was left overnight at 4 °C after adding equal volume of Acetone. Next day Centrifugation at 12,000×*g* for 20 min was applied to the solvent-coagulated EPS. Pure EPS powder was obtained after dialysis and lyophilization of purified EPS.

### Physiochemical characterization

#### Analysis of surface morphology (SEM–EDS and AFM)

Common physical properties of macromolecules can easily be identified from the study of their surface morphology [85, 86]. Scanning electron microscopy (SEM) and Atomic force microscopy (AFM) has been performed for determination of surface morphology. For executing SEM, a Gold Sputter Coater (DII 29030SCTR Smart Coater, JEOL, Tokyo, Japan) has been used to increase conductivity in EPS. FESEM (JEOl-JSM 7610FPlus) microscope has been used to observe the surface structure of the EPS. The accelerating voltage was 15 kV while performing SEM. Determination of carbon/nitrogen/oxygen/phosphorus/sulfur composition of the EPS has been executed by using an energy dispersive spectrometer (EDS) (X-Max-AZtec, Oxford Instruments).

For AFM 5 μL of the EPS solution was taken under the cover slip and air dried after making aqueous solution of EPS with 0.01 mg mL^−1^ concentration followed by vortexing well at room temperature. Then tapping mode of the NanoDrive Innova system AFM (diINNOVA, Bruker, Madison, WI, USA) was used for obtaining the images of EPS surface.

#### Analysis of monosaccharides composition of EPS

High pressure liquid chromatography has been performed for determination monosaccharide constituents of the purified BSP3 EPS. HPLC set up is assembled with LC-20AT Pump, multiple autosamplers (SIL-20A) fitted with a 20 μL loop, a UV detector (SPD-20AV) set to 210 and 290 nm, and a refractive index detector (RID-10A) with connecting the detectors. The LabSolution software Version 1.25 (Shimadzu, Kyoto, Japan) has been used for collecting HPLC data. A cation exchange column (Aminex HPX-87H) incorporated with a cation H^+^ microguard cartridge (Bio-Rad Laboratories, Hercules, CA, USA) has been used for isocratic analysis with water at 0.8 mL min^−1^ and 65 °C. For monosaccharide quantification, mannose, glucose, and galactose calibration curves were created using the sugar standards supplied by sigma Chile. The range of the sugar concentration was 200 to 10,000 μg mL^−1^.The calibration parameters for mannose, galactose and glucose were (147x – 26,559, R^2^ 0.997), (101x − 9566, R^2^ 0.999) and (140x − 6277, R^2^ 0.999), respectively.

#### Molecular weight determination of EPS

1100 HPLC system (Agilent Technologies, Santa Clara, USA) incorporated with Shodex columns (40, 300, 1000) has been used for performing gel permeation chromatography (GPC) for determining average molecular weight of the EPS. Pullulan (Sigma, St. Louis, United States) standards (0.3–700 kDa) has been used for GPC taking into consideration the polysaccharide nature of the EPS while maintaining following parameters using 0.1 M sodium nitrate solvent (sample = 10 μL, 0.1 g mL^−1^, Φ = 0.5 mL min^−1^; temperature = 50 °C).

### Structure determination

#### Fourier transform infrared spectroscopy (FTIR) and 1D NMR

The functional groups of the BSP3 EPS has been characterized using an FTIR-ATR spectrometer (Jasco-4000, Jasco Analytical Spain, Madrid, Spain) in transmittance mode. The samples were pressed into KBr pellets maintaining the ratio 1:90 before scanning. The scan range was 4000–500 cm^−1^ with 4 cm^−1^ resolution.

After dissolving the samples in D_2_O 1D NMR has been executed at 60 °C. Internal reference for ^1^H signals of water soluble samples chemical shift are expressed in d ppm relative to DSS as described in the Bruker manual. At 400 and 100 MHz ^1^H NMR spectrum has been recorded. In parts per million (ppm) chemical shifts (δ) are reported.

### Thermogravimetric analysis

A thermogravimetric analyzer (TGA) Cahn-Ventron 2000 (Cahn Scientific, Irvine, CA, USA) incorporated with microprocessor driven temperature-control unit has been used to determine the thermal stability of the BSP3 EPS. Aluminum sample pan has been used for placing the sample (approx. 5 mg) and the analysis was done maintaining the following parameters (temperature = 25–600 °C, heating rate 10 °C min^−1^, N_2_ gas flow rate 50 mL min^−1^.

### Analysis of the properties related to function of EPS

#### Antioxidant activity analysis

##### H_2_O_2_ scavenging activity

Following the steps mentioned by Ruch et al. [87], H_2_O_2_ scavenging activity has been analyzed with some modifications. 50 μL of EPS samples with 0.2, 0.5, 1, 2, and 5 mg mL^−1^ concentration has been prepared and 120 μL of 0.1 M phosphate buffer (pH 7.40) added to it. Then Mobi-Microplate Spectrophotometer (μ2 MicroDigital) has been used at 230 nm to record the absorbance of the samples. 30 μL of H_2_O_2_ solution (40 mM) has been added. After shaking vigorously reaction mixtures were incubated for 10 min at 30 °C. Then again, the absorbance of reaction mixture has been recorded at 230 nm.

1 $$\left[ {{1} - \left( {{\text{A}}_{{1}} - {\text{A}}_{{2}} } \right)/{\text{A}}_{0} } \right] \, \times {1}00.$$

Equation (1) has been used to calculating the H_2_O_2_ scavenging activity. Here A_1_ is the absorbance of the sample with H_2_O_2_, A_2_ is the absorbance of the sample without H_2_O_2_ and A_0_ is the absorbance of the distilled water. Positive control was Xanthan gum in this analysis.

##### ABTS free radical scavenging activity

Following the steps mentioned by Nitha et al. [88] 2,2′-azino-bis (3-ethylbenzothiazolin-6-sulfonic acid)/ABTS free radical scavenging activity has been also performed with some modifications. After dilution of ABTS at an absorbance of ~ 0.75 at 734 nm in pH 7.40 phosphate buffer, 180 μL of EPS samples with 0.2, 0.5, 1, 2, and 5 mg mL^−1^ concentration has been prepared. Then Mobi-Microplate Spectrophotometer (μ2 MicroDigital) has been used at 734 nm to record the absorbance of the samples. Then 20 μL of ABTS solution has been added. After allowing the reaction mixture to react for 5 min the absorbance of supernatant has been recorded at 734 nm.

2 $$\left[ {\left( {{\text{A}}_{0} - {\text{ A}}_{{1}} } \right)/{\text{A}}_{0} } \right] \, \times { 1}00.$$

Equation (2) has been used to calculating the ABTS RSA. Here A_1_ is the absorbance of the sample with ABTS, A_0_ is the absorbance of the sample without ABTS. Ascorbic acid was taken as positive control in this analysis.

##### FRAP activity

Following the steps mentioned by Benzie and Strain [89], the Ferric reducing Antioxidant Power Activity (FRAP) has been analyzed with some modifications using the FRAP assay kit (BioVision, MilPitas, USA). 10 μL of EPS samples with 0.2, 0.5, 1, 2, and 5 mg mL^−1^ concentration has been prepared. 19 µL of ferric chloride (FeCl_3_), 152 µL of FRAP assay buffer and 19 µL of FRAP probe was added to it. The absorbance of the mixture was measured at 594 nm in Mobi-Microplate Spectrophotometer (µ2 MicroDigital, Seoul, South Korea) after incubating at 30 °C for 60 min in dark condition.

3 $$\mathrm{B }\times \frac{D}{V}.$$

Equation (3) has been used to calculate the FRAP activity. Where B is the amount of ferrous ammonium sulfate from the standard curve (nmol), D is the dilution factor, and V is the volume of sample added to the reaction well (in µl). Positive control was Xanthan gum in this analysis. Calibration curve was calculated using different concentrations of ferrous standard provided in the kit.

#### Determination of flocculation activity

Following the method given by Pu et al. [90],with some modification the flocculation activity of BSP3 EPS has been analyzed. Xanthan gum has been used as control in this study. Kaolin suspension containing 1% CaCl2 (pH 7.0, 4 g L^−1^) has been prepared and mixed with different concentration EPS ranging from 5 to 100 mg L^−1^ n 1:1 v/v ratio. After stirring well, the mixture was left undisturbed for 10 min. Mobi-Microplate Spectrophotometer (μ2 MicroDigital) has been used to record the absorbance of the supernatant at 550 nm and Eq. (4) has been used in calculation of flocculating percentage:

4 $$\left[ {\left( {{\text{A}} - {\text{B}}} \right)/{\text{A}}} \right] \, \times {1}00,$$

where A and B is the absorbance of control supernatant and sample respectively.

#### Determination of emulsifying activity

Following the steps described by Cooper et al. [91], the study of the emulsifying activity has been performed with some modification. 1 mg mL^−1^ aqueous solution of EPS has been prepared and mixed with olive, sunflower, corn, canola, avocado, sesame and coconut oils maintaining the v/v Oil:EPS ratio of 3:2. The mixtures were vortexed for 2 min and at 24 h intervals the oil, emulsion and aqueous layer was measured. The emulsification index (E) has been calculated as [(volume of the emulsion layer × total volume^−1^) × 100]. The emulsifying property of the BSP3 EPS has been compared with Xanthan gum (Sigma) and Tween 20 (Sigma) taking into consideration that Xanthan gum is the most popular commercial EPS which is widely used in industries.

#### Determination of OHC and WHC

With little modification in the standard method described by Wang et al. [92], the oil holding capacity (OHC) of the BSP3 EPS has been determined. For this study 500 mg of lyophilized EPS has been mixed with 10 mL sunflower oil in cyclomixer. After allowing the samples to stand for 30 min at 37 °C with intermediate agitation at every 10 min, the samples were centrifuged at 3200 rpm for 25 min. Then decanting the centrifugation, the tube was weighted and OHC was calculated using the following Eq. (5).

5 $$\mathrm{\%OHC}=\frac{Oil\,bound\,weight (g)}{Initial\,sample\,weight (g)} \times 100.$$

Following the standard method given by Kumari et al. [93] the water holding capacity (WHC) of the BSP3 EPS has been determined. For this study 500 mg of lyophilized EPS has been mixed in 10 mL of water in cyclomixer for 1 min. After allowing the samples to stand for 30 min at 37 °C with intermediate shaking at every 10 min, the samples were centrifuged at 3200 rpm for 25 min. Then decanting the centrifugation, the tube was weighted and WHC was calculated using the following equation.

6 $$\mathrm{\%WHC}=\frac {Water\,bound\,weight (g)}{Initial\,sample\,weight (g)}\ \times 100.$$

Statistical program SigmaPlot 12.0 (Systat Software Inc., London, UK) has been used to calculate mean, SD, analysis of variance (ANNOVA) after performing OHC and WHC study of BSP3 EPS in three replicates. P value < 0.05 was considered using Bonferroni test. In both the experiments Xanthan gum has been used as positive control.

### Supplementary Information

**Additional file 1: Table S1A.** Heavy metal composition of the water sample collected from San Pedro hot spring (n = 2). **Table S1B.** Physicochemical parameters of the water sample collected from San Pedro hot spring (n = 2). **Table S1C.** Anions composition of the water sample collected from San Pedro hot spring (n = 2).

## Data Availability

All data generated or analysed during this study are included in this published article [and its Additional files].

## References

[1] Moldes AB, Vecino X, Cruz JM (2017). Nutraceuticals and food additives. Current developments in biotechnology and bioengineering.

[2] Faustino M, Veiga M, Sousa P, Costa E, Silva S, Pintado M (2019). Agro-food byproducts as a new source of natural food additives. Molecules..

[3] Ale EC, Perezlindo MJ, Pavón Y, Peralta GH, Costa S, Sabbag N (2016). Technological, rheological and sensory characterizations of a yogurt containing an exopolysaccharide extract from *Lactobacillus fermentum* Lf2, a new food additive. Food Res Int.

[4] Wang J, Salem DR, Sani RK (2019). Extremophilic exopolysaccharides: a review and new perspectives on engineering strategies and applications. Carbohydr Polym.

[5] Asgher M, Qamar SA, Iqbal HMN (2021). Microbial exopolysaccharide-based nano-carriers with unique multi-functionalities for biomedical sectors. Biologia (Bratisl).

[6] Shi Y, Huang J, Zeng G, Gu Y, Chen Y, Hu Y (2017). Exploiting extracellular polymeric substances (EPS) controlling strategies for performance enhancement of biological wastewater treatments: an overview. Chemosphere.

[7] Radchenkova N, Panchev I, Vassilev S, Kuncheva M, Dobreva S, Kambourova M (2015). Continuous cultivation of a thermophilic bacterium *Aeribacillus pallidus* 418 for production of an exopolysaccharide applicable in cosmetic creams. J Appl Microbiol.

[8] Llamas I, Mata JA, Tallon R, Bressollier P, Urdaci MC, Quesada E (2010). Characterization of the exopolysaccharide produced by *Salipiger mucosus* a3t, a halophilic species belonging to the alphaproteobacteria, isolated on the spanish mediterranean seaboard. Mar Drugs.

[9] Llamas I, Amjres H, Mata JA, Quesada E, Béjar V (2012). The potential biotechnological applications of the exopolysaccharide produced by the halophilic bacterium *Halomonas almeriensis*. Molecules.

[10] Rossi F, De Philippis R (2015). Role of cyanobacterial exopolysaccharides in phototrophic biofilms and in complex microbial mats. Life.

[11] Fan Y, Wang J, Gao C, Zhang Y, Du W (2020). A novel exopolysaccharide-producing and long-chain *n*-alkane degrading bacterium *Bacillus licheniformis* strain DM-1 with potential application for in-situ enhanced oil recovery. Sci Rep.

[12] Gupta PL, Rajput M, Oza T, Trivedi U, Sanghvi G (2019). Eminence of microbial products in cosmetic industry. Nat Prod Bioprospect.

[13] Wang Z, Wu J, Zhu L, Zhan X (2017). Characterization of xanthan gum produced from glycerol by a mutant strain *Xanthomonas campestris* CCTCC M2015714. Carbohydr Polym.

[14] Rehm BHA, Valla S (1997). Bacterial alginates: biosynthesis and applications. Appl Microbiol Biotechnol.

[15] Gongi W, Cordeiro N, Pinchetti JLG, Ben OH (2022). Functional, rheological, and antioxidant properties of extracellular polymeric substances produced by a thermophilic cyanobacterium *Leptolyngbya* sp. J Appl Phycol..

[16] Schiano Moriello V, Lama L, Poli A, Gugliandolo C, Maugeri TL, Gambacorta A (2003). Production of exopolysaccharides from a thermophilic microorganism isolated from a marine hot spring in flegrean areas. J Ind Microbiol Biotechnol.

[17] Xu Z, Guo Q, Zhang H, Wu Y, Hang X, Ai L (2018). Exopolysaccharide produced by *Streptococcus thermophiles* S-3: molecular, partial structural and rheological properties. Carbohydr Polym.

[18] Zhao N, Yu T, Yan F (2023). Probiotic role and application of thermophilic *Bacillus* as novel food materials. Trends Food Sci Technol.

[19] Gupta GN, Srivastava S, Khare SK, Prakash V (2014). Extremophiles: an overview of microorganism from extreme environment. Int J Agric Environ Biotechnol.

[20] Jin M, Gai Y, Guo X, Hou Y, Zeng R (2019). Properties and applications of extremozymes from deep-sea extremophilic microorganisms: a mini review. Mar Drugs.

[21] van den Burg B (2003). Extremophiles as a source for novel enzymes. Curr Opin Microbiol.

[22] Beeler E, Singh OV (2016). Extremophiles as sources of inorganic bio-nanoparticles. World J Microbiol Biotechnol.

[23] Seckbach J, Oren A (2000) Extremophilic microorganisms as candidates for extraterrestrial life. In: Hoover RB, editor. p. 89–95. 10.1117/12.411613.

[24] Kristjnsson JK, Hreggvidsson GO (1995). Ecology and habitats of extremophiles. World J Microbiol Biotechnol.

[25] Zgonik V, Mulec J, Eleršek T, Ogrinc N, Jamnik P, Ulrih NP (2021). Extremophilic microorganisms in central Europe. Microorganisms.

[26] Poli A, Finore I, Romano I, Gioiello A, Lama L, Nicolaus B (2017). Microbial diversity in extreme marine habitats and their biomolecules. Microorganisms.

[27] Kambourova M, Radchenkova N, Tomova I, Bojadjieva I (2016). Thermophiles as a promising source of exopolysaccharides with interesting properties. Biotechnology of extremophiles.

[28] Flemming HC (2016). Eps—then and now. Microorganisms.

[29] Webster GK, Craig RA, Pommerening CA, Acworth IN (2012). Selection of pharmaceutical antioxidants by hydrodynamic voltammetry. Electroanalysis.

[30] Wang J, Salem DR, Sani RK (2021). Two new exopolysaccharides from a thermophilic bacterium *Geobacillus* sp. WSUCF1: characterization and bioactivities. N Biotechnol..

[31] Banerjee A, Rudra SG, Mazumder K, Nigam V, Bandopadhyay R (2018). Structural and functional properties of exopolysaccharide excreted by a novel *Bacillus anthracis* (strain PFAB2) of hot spring origin. Indian J Microbiol.

[32] Banerjee A, Breig SJM, Gómez A, Sánchez-Arévalo I, González-Faune P, Sarkar S (2022). Optimization and characterization of a novel exopolysaccharide from *Bacillus haynesii* camb6 for food applications. Biomolecules.

[33] Orellana R, Macaya C, Bravo G, Dorochesi F, Cumsille A, Valencia R (2018). Living at the frontiers of life: extremophiles in Chile and their potential for bioremediation. Front Microbiol..

[34] Scherson RA, Thornhill AH, Urbina-Casanova R, Freyman WA, Pliscoff PA, Mishler BD (2017). Spatial phylogenetics of the vascular flora of Chile. Mol Phylogenet Evol.

[35] Sabando C, Ide W, Rodríguez-Díaz M, Cabrera-Barjas G, Castaño J, Bouza R (2020). A novel hydrocolloid film based on pectin, starch and *Gunnera tinctoria* and *Ugni molinae* plant extracts for wound dressing applications. Curr Top Med Chem.

[36] Liang X. Structural characterization and bioactivity of exopolysaccharide synthesized by *Geobacillus* sp Ts3-9 isolated from radioactive radon hot spring. Adv Biotechnol Microbiol. 2017;4(2). 10.19080/AIBM.2017.04.555635.

[37] Synytsya A, Novak M (2014). Structural analysis of glucans. Ann Transl Med..

[38] Yao HYY, Wang JQ, Yin JY, Nie SP, Xie MY (2021). A review of nmr analysis in polysaccharide structure and conformation: progress, challenge and perspective. Food Res Int.

[39] Jana UK, Kango N (2020). Characteristics and bioactive properties of mannooligosaccharides derived from agro-waste mannans. Int J Biol Macromol.

[40] Gómez-Miranda B, Prieto A, Leal JA, Ahrazem O, Jiménez-Barbero J, Bernabé M (2003). Differences among the cell wall galactomannans from *Aspergillus wentii* and *Chaetosartorya chrysella* and that of *Aspergillus fumigatus*. Glycoconj J.

[41] Kobayashi H, Suzuki J, Tanaka S, Kiuchi Y, Oyamada H, Iwadate N (1997). Structure of a cell wall mannan from the pathogenic yeast, *Candida catenulata*: assignment of 1H nuclear magnetic resonance chemical shifts of the inner α-1,6-linked mannose residues substituted by a side chain. Arch Biochem Biophys.

[42] Kobayashi H, Watanabe M, Komido M, Matsuda K, Ikeda-Hasebe T, Suzuki M (1995). Assignment of 1H and 13C NMR chemical shifts of a d-mannan composed of α-(1 → 2) and α-(1 → 6) linkages obtained from *Candida kefyr* IFO 0586 strain. Carbohydr Res.

[43] Feng L, Yin J, Nie S, Wan Y, Xie M (2018). Structure and conformation characterization of galactomannan from seeds of *Cassia obtusifolia*. Food Hydrocoll.

[44] Li Y, Liu H, Shi Y, Yan Q, You X, Jiang Z (2020). Preparation, characterization, and prebiotic activity of manno-oligosaccharides produced from cassia gum by a glycoside hydrolase family 134 β-mannanase. Food Chem..

[45] Casillo A, Fabozzi A, Russo Krauss I, Parrilli E, Biggs CI, Gibson MI (2021). Physicochemical approach to understanding the structure, conformation, and activity of mannan polysaccharides. Biomacromol.

[46] Rugar D, Hansma P (1990). Atomic force microscopy. Phys Today.

[47] Cordell D, Unsworth MJ, Díaz D (2018). Imaging the laguna del maule volcanic field, central chile using magnetotellurics: evidence for crustal melt regions laterally-offset from surface vents and lava flows. Earth Planet Sci Lett.

[48] Taran Y, Kalacheva E (2020). Acid sulfate-chloride volcanic waters; Formation and potential for monitoring of volcanic activity. J Volcanol Geoth Res.

[49] Lahsen A (1988). Chilean geothermal resources and their possible utilization. Geothermics.

[50] Kambourova M, Mandeva R, Dimova D, Poli A, Nicolaus B, Tommonaro G (2009). Production and characterization of a microbial glucan, synthesized by *Geobacillus tepidamans* V264 isolated from Bulgarian hot spring. Carbohydr Polym.

[51] Sardari RRR, Kulcinskaja E, Ron EYC, Björnsdóttir S, Friðjónsson ÓH, Hreggviðsson GÓ (2017). Evaluation of the production of exopolysaccharides by two strains of the thermophilic bacterium *Rhodothermus marinus*. Carbohydr Polym.

[52] Feng F, Zhou Q, Yang Y, Zhao F, Du R, Han Y (2018). Characterization of highly branched dextran produced by Leuconostoc citreum B-2 from pineapple fermented product. Int J Biol Macromol.

[53] Wang Y, Li C, Liu P, Ahmed Z, Xiao P, Bai X (2010). Physical characterization of exopolysaccharide produced by *Lactobacillus plantarum* KF5 isolated from Tibet Kefir. Carbohydr Polym.

[54] Jana UK, Kango N (2020). Characteristics and bioactive properties of mannooligosaccharides derived from agro-waste mannans. Int J Biol Macromol.

[55] Arciola CR, Campoccia D, Gamberini S, Donati ME, Pirini V, Visai L (2005). Antibiotic resistance in exopolysaccharide-forming *Staphylococcus epidermidis* clinical isolates from orthopaedic implant infections. Biomaterials.

[56] Joyce JG, Abeygunawardana C, Xu Q, Cook JC, Hepler R, Przysiecki CT (2003). Isolation, structural characterization, and immunological evaluation of a high-molecular-weight exopolysaccharide from Staphylococcus aureus. Carbohydr Res.

[57] Marshall VM, Rawson HL (1999). Effects of exopolysaccharide-producing strains of thermophilic lactic acid bacteria on the texture of stirred yoghurt. Int J Food Sci Technol.

[58] Nicolaus B, Kambourova M, Oner ET (2010). Exopolysaccharides from extremophiles: from fundamentals to biotechnology. Environ Technol.

[59] Lee J, Cho YS, Jung H, Choi I (2018). Pharmacological regulation of oxidative stress in stem cells. Oxid Med Cell Longev.

[60] Kusumawati I, Indrayanto G (2013). Natural antioxidants in cosmetics.

[61] Maraveas C, Bayer IS, Bartzanas T (2021). Recent advances in antioxidant polymers: from sustainable and natural monomers to synthesis and applications. Polymers (Basel).

[62] Hu C, You G, Liu J, Du S, Zhao X, Wu S (2021). Study on the mechanisms of the lubricating oil antioxidants: experimental and molecular simulation. J Mol Liq..

[63] Dalton TP, Shertzer HG, Puga A (1999). Regulation of gene expression by reactive oxygen. Annu Rev Pharmacol Toxicol.

[64] Celestino MT, Magalhães de UO, Fraga AGM, Carmo do FA, Lione V, Castro HC (2012). Rational use of antioxidants in solid oral pharmaceutical preparations. Braz J Pharm Sci..

[65] Williams GM, Iatropoulos MJ, Whysner J (1999). Safety assessment of butylated hydroxyanisole and butylated hydroxytoluene as antioxidant food additives. FCT.

[66] Okubo T, Yokoyama Y, Kano K, Kano I (2004). Molecular mechanism of cell death induced by the antioxidant tert-butylhydroxyanisole in human monocytic leukemia u937 cells. Biol Pharm Bull.

[67] Hirose M (1998). Carcinogenicity of antioxidants bha, caffeic acid, sesamol, 4-methoxyphenol and catechol at low doses, either alone or in combination, and modulation of their effects in a rat medium-term multi-organ carcinogenesis model. Carcinogenesis.

[68] Bibi A, Xiong Y, Rajoka MSR, Mehwish HM, Radicetti E, Umair M (2021). Recent advances in the production of exopolysaccharide (eps) from Lactobacillus spp. and its application in the food industry: a review. Sustainability..

[69] Alizadeh-Sani M, Hamishehkar H, Khezerlou A, Azizi-Lalabadi M, Azadi Y, Nattagh-Eshtivani E (2018). Bioemulsifiers derived from microorganisms: applications in the drug and food industry. Adv Pharm Bull..

[70] Song B, Zhu W, Song R, Yan F, Wang Y (2019). Exopolysaccharide from Bacillus vallismortis WF4 as an emulsifier for antifungal and antipruritic peppermint oil emulsion. Int J Biol Macromol.

[71] Gupta BS, Ako JE (2005). Application of guar gum as a flocculant aid in food processing and potable water treatment. Eur Food Res Technol..

[72] Kurniawan SB, Imron MF, Chik CENCE, Owodunni AA, Ahmad A, Alnawajha MM (2022). What compound inside biocoagulants/bioflocculants is contributing the most to the coagulation and flocculation processes?. Sci Total Environ..

[73] Gupta A, Kumar M, Sharma R, Tripathi R, Kumar V, Thakur IS (2023). Screening and characterization of bioflocculant isolated from thermotolerant *Bacillus* sp. ISTVK1 and its application in wastewater treatment. Environ Technol Innov..

[74] Okaiyeto K, Nwodo U, Mabinya L, Okoh A (2013). Characterization of a bioflocculant produced by a consortium of halomonas sp. Okoh and Micrococcus sp. Leo. Int J Environ Res Public Health..

[75] Mathivanan K, Chandirika JU, Vinothkanna A, Govindarajan RK, Meng D, Yin H (2021). Characterization and biotechnological functional activities of exopolysaccharides produced by *Lysinibacillus fusiformis* kmntt-10. J Polym Environ.

[76] Trabelsi I, Ktari N, Triki M, Bkhairia I, Ben Slima S, Sassi Aydi S (2018). Physicochemical, techno-functional, and antioxidant properties of a novel bacterial exopolysaccharide in cooked beef sausage. Int J Biol Macromol.

[77] Devi PB, Kavitake D, Shetty PH (2016). Physico-chemical characterization of galactan exopolysaccharide produced by *Weissella confusa* KR780676. Int J Biol Macromol.

[78] Insulkar P, Kerkar S, Lele SS (2018). Purification and structural-functional characterization of an exopolysaccharide from *Bacillus licheniformis* PASS26 with in-vitro antitumor and wound healing activities. Int J Biol Macromol.

[79] Mohanasrinivasan V, Mishra M, Paliwal JS, Singh SKR, Selvarajan E, Suganthi V (2014). Studies on heavy metal removal efficiency and antibacterial activity of chitosan prepared from shrimp shell waste. 3 Biotech..

[80] Molina Olivera MG, Rivera Bravo DP, Salesianos Impresores S.A. Compendio Normativo de los servicios sanitarios: agua potable y saneamiento. 2018. https://www.siss.gob.cl/586/articles-16991_recurso_1.pdf.

[81] Fierro P, Tapia J, Bertrán C, Acuña C, Vargas-Chacoff L (2021). Assessment of heavy metal contamination in two edible fish species and water from north patagonia estuary. Appl Sci.

[82] Tamura K, Stecher G, Kumar S (2021). MEGA11: molecular evolutionary genetics analysis version 11. Mol Biol Evol.

[83] Tamura K (1993). Estimation of the number of nucleotide substitutions in the control region of mitochondrial DNA in humans and chimpanzees. Mol Biol Evol.

[84] Rimada PS, Abraham AG (2003). Comparative study of different methodologies to determine the exopolysaccharide produced by kefir grains in milk and whey. Lait.

[85] Patnaik SS, Bunning TJ, Adams WW, Wang J, Labes MM (1995). Atomic force microscopy and high-resolution scanning electron microscopy study of the banded surface morphology of hydroxypropyl cellulose thin films. Macromolecules.

[86] Malkin AJ, Kuznetsov YuG, McPherson A (1999). In situ atomic force microscopy studies of surface morphology, growth kinetics, defect structure and dissolution in macromolecular crystallization. J Cryst Growth..

[87] Ruch RJ, Cheng S, Klaunig JE (1989). Prevention of cytotoxicity and inhibition of intercellular communication by antioxidant catechins isolated from Chinese green tea. Carcinogenesis..

[88] Nitha B, De S, Adhikari SK, Devasagayam TPA, Janardhanan KK (2010). Evaluation of free radical scavenging activity of morel mushroom, *Morchella esculenta* mycelia: a potential source of therapeutically useful antioxidants. Pharm Biol.

[89] Benzie IFF, Strain JJ (1996). The ferric reducing ability of plasma (FRAP) as a measure of “antioxidant power”: the frap assay. Anal Biochem.

[90] Pu L, Zeng YJ, Xu P, Li FZ, Zong MH, Yang JG (2020). Using a novel polysaccharide BM2 produced by Bacillus megaterium strain PL8 as an efficient bioflocculant for wastewater treatment. Int J Biol Macromol.

[91] Cooper DG, Goldenberg BG (1987). Surface-active agents from Two *Bacillus* species. Appl Environ Microbiol.

[92] Wang JC, Kinsella JE (1976). Functional properties of novel proteins: alfalfa leaf protein. J Food Sci.

[93] Kumari S, Kumar Annamareddy SH, Abanti S, Kumar RP (2017). Physicochemical properties and characterization of chitosan synthesized from fish scales, crab and shrimp shells. Int J Biol Macromol.

