# Supplementary material for: Characterization of Chilean hot spring-origin Staphylococcus sp. BSP3 produced exopolysaccharide as biological additive
Source: Nat Prod Bioprospect. 2024 Feb 4;14(1):15. doi: 10.1007/s13659-024-00436-0 (PMC10838260; doi:10.1007/s13659-024-00436-0)
Supplement: Supplementary file 1 — Additional file 1: Table S1A. Heavy metal composition of the water sample collected from San Pedro hot spring (n = 2). Table S1B. Physicochemical parameters of the water sample collected from San Pedro hot spring (n = 2). Table S1C. Anions composition of the water sample collected from San Pedro hot spring (n = 2). [file 13659_2024_436_MOESM1_ESM.docx]

| **Heavy Metal** | **Detection Limit (μg L^−1^)** | **Sample (mg mL^-1^)** |
| --- | --- | --- |
| Al | 28 | 0.24 ± 0.00 |
| As | 0.04 | 0.002 ± 0.00 |
| Cd | 2.8 | <0.02 |
| Cu | 4.5 | 0.07 ± 0.00 |
| Cr | 5.4 | 0.009 ± 0.00 |
| Fe | 4.3 | 2.20 ± 0.50 |
| Mn | 1.6 | 4.85 ± 0.00 |
| Mg | 2.2 | 19.98 ± 0.76 |
| Hg | 0.06 | < 0.0005 |
| Ni | 8 | 0.23 ± 0.02 |
| Pb | 13 | 0.26 ± 0.05 |
| Zn | 3.3 | 0.09 ± 0.01 |

**Supplementary Table S1A:** Heavy metal composition of the water sample collected from San Pedro hot spring (n=2)

**Supplementary Table S1B:** Physicochemical parameters of the water sample collected from San Pedro hot spring (n=2)

**Supplementary Table S1C:** Anions composition of the water sample collected from San Pedro hot spring (n=2)

| **Parameter** | **Unit** | **Sample** |
| --- | --- | --- |
| Conductivity | mS/cm | 37.74 ± 0.24 |
| Total alkalinity | mg/L CaCO3 | 442.9 ± 1.40 |
| Dissolve solid | g/L | 30.80 ± 3.44 |
| Suspended Solid | mg/L | 15.8 ± 1.00 |
| Turbidity | NTU | 10.70 ± 0.21 |

| **Anions** | **Sample (mg mL^-1^)** |
| --- | --- |
| CN^-^ | < 0.005 |
| Cl^-^ | 13.93 ± 0.07 |
| NO_3_^-^ | 5.34 ± 0.29 |
| NO_2_^-^ | 0.071 ± 0.005 |
| SO_4_^2-^ | 439.7 ± 1.80 |
| F^-^ | 1.3 ± 0.00 |
